# Assessment of the effectiveness of art drawing for children with ASD and prediction of risk factors

**DOI:** 10.1097/MD.0000000000046881

**Published:** 2026-01-02

**Authors:** Jinghong Yao, Qiushi Wang, Yang Qiao, Ying Cai, Heyong Shen

**Affiliations:** a College of Humanities and Education, Wuyi University, Wuyishan City, Fujian Province, China; b Political Public Course Teaching and Research Center Shenzhen Campus, Jinan University, Shenzhen City, Guangdong Province, China; c Office of Student Affairs, Shandong University of Science and Technology, Qingdao City, Shandong Province, China; d Student Development Guidance Center, Division of Student Affairs, Harbin Engineering University, Harbin City, Heilongjiang Province, China; e Institute of Psychoanalysis, City University of Macau, Macau, China.

**Keywords:** anxiety, artistic painting, ASD

## Abstract

Autism spectrum disorder (ASD) is a recognized public health issue with unknown origins. This study explored the use of art therapy to address anxiety and behavioral issues in ASD, focusing on individualized treatment and multi-method assessment. A total of 131 children with ASD, aged 3 to 8, were divided into a control and a painting group. Both received a drawing book for home use. The painting group underwent 3 types of drawing therapy, and their effectiveness was analyzed using logistic regression, with anxiety levels as the dependent variable. The painting group showed reduced anxiety during preparation and pre-anesthetic visits compared to the control group. The control group had a higher rate of poor treatment adherence. House-tree-person drawing therapy was linked to reduced anxiety, while mandala and group drawing therapies showed no significant (*P* > .05) correlation with anxiety levels. Group therapy effectiveness increased (*P* < .05) with participant numbers. Art drawing can significantly reduce anxiety in children with ASD and improve treatment adherence. House-tree-person drawing is useful for anxiety screening, while mandala and group therapies offer personalized treatment approaches.

## 1. Introduction

An unfamiliar environment often triggers substantial stress in children, eliciting a range of physiological and psychological responses.^[1]^ Among pediatric populations, anxiety is particularly prevalent in those with autism spectrum disorders (ASDs), with reported rates between 40% and 65%.^[2,3]^ Such anxiety may emerge as soon as the child is informed of the need for hospitalization and typically intensifies during the hospital stay.^[1]^ Elevated anxiety negatively influences both physical and psychological functioning, interferes with treatment adherence, prolongs recovery, and can hinder cooperation in self-care. Heightened sensitivity to environmental stimuli, episodes of delirium, and abrupt behavioral changes are also recognized manifestations of treatment-related stress in children.^[4–6]^ If unmanaged, this anxiety may contribute to long-term psychological complications.^[4]^ Conversely, reducing anxiety can facilitate more effective treatment, accelerate recovery and rehabilitation, decrease medication requirements, improve pain tolerance, and shorten hospital stays.^[6]^

Preparing children for hospitalization therefore requires targeted efforts to enhance their physical and emotional readiness and to minimize anxiety. Distraction-based approaches represent a widely used nursing intervention for this purpose.^[1]^ Strategies for reducing anxiety can be broadly categorized into pharmacological and non-pharmacological modalities.^[5]^ Non-pharmacological techniques, which carry fewer adverse effects, may be used independently or alongside other treatments. These include enjoyable activities such as playing games, drawing, watching films, engaging in computer games, and storytelling. Through these activities, children interact with peers, symbolically process fears, express emotions, and collaborate more effectively with healthcare providers, thereby supporting their physical, cognitive, and emotional development.^[7]^ Despite their potential, research examining the impact of such interventions on children’s mental health remains limited.

Previous studies have demonstrated that enjoyable activities can lessen children’s anxiety,^[8]^ and game-based interventions have shown similar benefits.^[9]^ However, even with the provision of toys or access to playrooms, some residual anxiety typically persists.^[8,9]^ Interactive play, in particular, has been shown to enhance communication and social skills, allowing children to articulate emotions, gain new perspectives, and adopt more adaptive coping strategies; thus, interactive games were selected as one of the interventions in the present study.^[10,11]^

Art therapy is another widely applied non-pharmacological method for managing behavioral and emotional difficulties, particularly stress and aggression. As one of its primary modalities, drawing encourages spontaneous image creation and allows children to express themselves through non-verbal channels.^[11]^ The aim is not to teach artistic skills, but to provide a medium through which children can freely communicate their feelings, needs, and experiences using colors and lines. Drawing bridges internal emotions and external reality, enabling the expression of repressed thoughts in a safe manner. Through participation in art-making, individuals externalize their inner world, regulate emotions, and strengthen psychological resilience.^[12–14]^ Numerous studies support the therapeutic value of drawing; for example, Zarezadeh Kheibari et al^[11]^ reported that group art therapy reduced anxiety in orphans, and Safaria et al found that art therapy alleviated anxiety among victims of bullying.^[15]^ Given that nurses spend more time with children than other healthcare professionals, they are particularly well positioned to apply such non-pharmacological approaches to reduce anxiety.^[16]^

Considering the limited comparative evidence on different distraction techniques – particularly interactive games versus drawing – for anxiety reduction in hospitalized children, the present study aimed to evaluate the effects of these 2 interventions on pediatric anxiety during hospitalization.

## 2. Materials and methods

### 2.1. Design and participants

This retrospective study was approved by the Ethics Committee of the City University of Macau. Medical records of children with ASD who received treatment between January 2018 and April 2023 were reviewed. A total of 108 children, aged 3 to 12 years, met the inclusion criteria and were included in the analysis.

Based on the recorded intervention type, participants were categorized into a drawing therapy group (n = 55) and a control group (n = 53). The grouping reflected the treatment each child had previously received during the clinical period. To minimize selection bias, children were matched retrospectively according to demographic and clinical characteristics, including age, gender, and previous hospitalization history.

The inclusion criteria were as follows: children aged 3 to 12 years; diagnosed with ASD and able to engage in interactive activities; capable of simple verbal communication; complete parental consent documented in the medical record; absence of other mental or behavioral comorbidities; and at least one parent accompanied the child during treatment.

### 2.2. Study procedures

All participants underwent an eligibility assessment, and following recruitment, basic information and characteristics of the children and their parents were collected. This included birth dates, gender, the child’s temperament (emotional, activity, sociability, and impulsiveness, as measured by the emotionality, activity, sociability, and impulsivity (temperament tool)),^[9]^ the parents’ educational level, marital status, whether the child was an only child, living conditions, and time spent alone, among other details. Additionally, general information regarding the child’s preferences was gathered. Parents also received verbal instructions regarding the drawing therapy intervention prior to the child’s treatment. A week before the child’s hospitalization, a drawing book and detailed reading prompts were mailed to the homes of the study participants. Parents were instructed not to withhold information about the treatment from their children but instead to help them understand the basics of drawing through the use of the picture book. Under the guidance of their parents, children were encouraged to look through the series of images to familiarize themselves with the characters depicted in the book and the environment of the therapy room, thus forming a preliminary impression of drawing. Subsequently, the children and adults would view these images together. During this process, parents would ask for the child’s opinions on the images and pose questions to the child, particularly focusing on aspects such as what they found appealing, what the images resembled, why certain places were depicted in a specific way, and how certain parts were drawn. This approach was designed to encourage greater child involvement in the joint drawing sessions.

### 2.3. Data collection

The therapeutic effects of the art drawing intervention on children with ASD were assessed at 2 main time points: before the intervention (baseline) and 6 months after the intervention (follow-up).

At baseline, anxiety during clinical procedures was evaluated using the modified Yale Preoperative Anxiety Scale-Short Form (mYPAS-SF), which measures situational anxiety in children through 5 domains: engagement in activities (4 items), communication (6 items), emotional expression (4 items), vocalization (4 items), and parental use (4 items), totaling 22 items. Each item is scored on a 1 to 4 or 1 to 6 scale depending on the domain, with higher scores indicating greater anxiety. The total mYPAS-SF score is calculated as the sum of domain scores divided by the maximum possible score for that domain. The scale has demonstrated strong validity and reliability in previous studies,^[17–19]^ and the Cronbach alpha in the present study was 0.85.

At the 6-month follow-up, overall psychological well-being was assessed using the self-rating anxiety scale (SAS), self-rating depression scale, and the short form health survey. These instruments were used to evaluate changes in anxiety, depressive symptoms, and quality of life among the participating children. The SAS and self-rating depression scale are widely used self-report scales adapted for use with children under parental supervision, while the short form health survey provides a broad measure of physical and psychological health.

## 2.4. Intervention therapy

For the drawing group, the therapeutic process began with the therapist establishing an appropriate therapeutic relationship with the child and providing familiar items to the child 20 to 30 minutes before entering the therapy room. The children were allowed to play with toys (playdough, building blocks, and puzzles) in the presence of a parent to help regulate their emotions. Following this, the therapist initiated an interactive engagement with the child, establishing a connection before providing the necessary drawing tools, which included A4 paper, pencils, erasers, and a selection of colored pencils. The children were then encouraged to draw and color according to their own desires. During the process of drawing and playing, the children were able to communicate with each other in a friendly environment and express their thoughts. The anxiety levels of the patients were measured both before and after being taken to the therapy room. In contrast, the control group received standard medical care without the additional art drawing intervention.

## 2.5. Self-assessment scale for anxiety (SAS)

The SAS is a self-assessment scale with 20 entries, including 5 reverse-scored entries and 15 positive-scored entries, scored on a 4-point scale from 1 to 4. All entries are summed to obtain a total score, which is multiplied by 1.25 times to obtain the SAS standard score. When assessing, screening or conducting large-scale surveys on anxiety disorders in China’s psychiatry community, we refer to Zung severity grading standard, respectively, and use the SAS score > 50 as the cutoff point for the severity of anxiety symptoms, and use the Chinese national norm score, the higher the score, the more severe the anxiety state, which is in line with Zung method.^[20–23]^

## 2.6. Implementation of drawing therapy

The whole process of house-tree-person (S-HTP) drawing therapy is as follows: Tools: a piece of A4 white paper (210 mm × 297 mm), a 2B pencil, an eraser, a writing board; Instruction: please draw a house, a tree, a person, and anything you want to draw in a picture. There is no time limit; Note: The test must be completed by the individual. The patient should be informed that the ability to draw is not important and that they should only complete the drawing carefully. They cannot copy or use other tools. The researcher cannot give any evaluation or hints.

The whole process of mandala drawing therapy is as follows: Preparation: Select art materials, relax, and set intentions; Center dot: Draw a central dot on the canvas as the starting point of the mandala; Structure building: Create the basic structure of the mandala with circles and geometric shapes around the center; Detailing: Enrich the mandala with colors, spirals, patterns, or symbols; Reflection and sharing: Reflect on the experience after completion and consider sharing with others or keeping it personal.

The whole process of group drawing art therapy is as follows: Group formation: Establish the group, explain the purpose, and create a supportive atmosphere; Warm-up activities: Engage in activities to help members relax and prepare to participate; Theme selection: Decide on a theme or direction for the group’s drawing; Collaborative creation: Group members contribute to the artwork, expressing individual thoughts; and Discussion and reflection: Share the meaning of the creative process and artwork to foster personal and group growth.

## 2.7. Evaluation of efficacy

Inter-rater reliability (Kappa coefficient) was used in this study Kappa coefficient values ranged from 0.752 to 1.000. The *R*-value of retest reliability for patient retest was [0.710, 0.857]. This indicates stable and reliable results for drawing features. In this study, validity was used to indicate the effect of drawing treatment. Correlation analyses were carried out between the anxiety data of the patients identified by the drawing treatment and the SAS scale (with the results of the SAS scale as the “gold standard”), and the correlation coefficient was the coefficient of validity.

## 2.8. Univariate analysis

Considering the number of cases and the number of drawings (anxiety dimension) in this study, univariate analyses were performed according to statistical theory. A χ^2^ test was performed on all 26 items in the drawing and control groups. The results showed that there were 12 different characteristics between the drawing and control groups. In order to capture all significant factors, characteristics with a *P*-value ≤ .15 were included as independent variables in logistic regression analyses.

## 2.9. Data analysis

Data analysis was conducted using SPSS 18.0. Descriptive statistics summarized participant demographics. Measurement data were expressed as mean ± standard deviation (x̄ ± s) and compared using *t*-tests, while categorical data were analyzed with χ^2^ or Fisher exact tests. A *P*-value < .05 was considered statistically significant. Before recruitment, a sample size estimation using G*Power 3.1 (two-tailed test, α = 0.05, power = 0.80, medium effect size *d* = 0.5) indicated a minimum of 102 participants. To ensure adequate power and account for attrition, 108 children were enrolled. χ^2^ tests and one-way analysis of variance (ANOVA) assessed baseline homogeneity and intervention effects. Paired *t*-tests compared pre- and post-intervention outcomes within groups. Multivariate logistic regression examined factors influencing treatment effectiveness, controlling for potential confounders.

## 3. Results

### 3.1. Univariate analysis of variance between the 2 groups

Of the 108 participating children, 59.3% were boys and 40.7% were girls. The mean age of all participants was 7.00 ± 2.60 years. The mean ages of the control and painting groups were 7.32 ± 2.75 and 7.64 ± 2.63 years, respectively. One-way ANOVA showed no significant differences between the groups in mean age, duration of treatment, or number of treatments. χ^2^ tests also revealed no significant differences between the 2 groups in terms of residence, gender, parents’ educational level, history of hospitalization or surgery, and economic status (Table 1). Paired *t*-tests showed no significant differences in the control group in all domains and mean total anxiety scores before and after the study; however, comparing the pre and post scores of the intervention group by paired *t*-tests, the difference between the mean total anxiety scores of the painting group and the control group was statistically significant (*P* = .001). Whereas in the comparison of mean scores in all domains of anxiety, there was no significant difference in all domains in the control group except for the domain of emotional expression (*P* = .007) in the drawing group (Tables 2–4). The mean score of preoperative anxiety was 57.45 ± 17.93 in the control group and 50.76 ± 19.20 in the drawing group. The results of one-way ANOVA showed that there was no statistically significant difference in the *t*-test between the groups (*P* = .058). The mean pretreatment anxiety scores in the control and drawing groups were 56.50 ± 15.63 and 49.91 ± 13.21, respectively. one-way ANOVA results showed a statistically significant difference between the 2 groups (*P* < .001). Before and after the intervention, the mean value of the difference in total anxiety scores was 0.95 ± 11.29 in the control group and 8.56 ± 16.69 in the painting group. The results of one-way ANOVA showed a significant difference between the groups (*P* = .010) (Table 5). Post hoc Scheffe test showed that the mean anxiety score of the drawing group was significantly lower than that of the control group.

**Table 1:** Baseline characteristics of the study participants.

Group	Painting	Control	*P*-value
Demographic data			
Age mean length (d) of hospital stay	7.64 ± 2.33	2.75 ± 7.32	.53
Gender (%)			
Male	33 (60)	31 (58.5)	.59
Female	22 (40)	22 (41.5)	
Residence (%)			
Rural	19 (34.5)	17 (32.7)	.39
Urban	36 (65.5)	35 (67.3)	
Time at hospitalization n (%)			
The day before the day of surgery	36 (66.7)	17 (32.1)	.57
The night before surgery	7 (13)	16 (30.2)	
The morning before surgery	11 (20.4)	20 (37.7)	
Previous hospitalization n (%)			
Yes	22 (40)	23 (43.4)	.16
No	33 (60)	30 (56.6)	
Number of hospitalizations n (%)			
Once	11 (73.3)	8 (44.4)	.10
Twice	1 (6.7)	7 (38.9)	
More than twice	3 (20)	3 (16.7)	
Previous surgery n (%)			
Yes	9 (17)	10 (20)	.94
No	44 (83)	40 (80)	
Number of surgeriesn (%)			
Once	6 (75)	3	.00
Twice	2 (25)	(60)	
Going to participation n (%)			
Yes	10 (31.3)	12 (29.3)	.17
No	22 (68.8)	29 (70.7)	
Father education n (%)			
Iliterate	1 (1.8)	1 (1.9)	.08
Under high school	39 (70.9)	25 (47.2)	
High school completion	13 (23.6)	17 (32.1)	
Academic	2 (3.6)	10 (18.9)	
Mother education n (%)			
Iliterate	3 (5.5)	1 (1.9)	.13
Under high school	36 (65.5)	25 (47.2)	
High school completion	12 (21.8)	16 (30.2)	
Academic	4 (7.3)	11 (20.8)	
Economic status n (%)			
Poor	15 (28.3)	3 (5.7)	.07
Average	35 (66)	41 (77.4)	
Good	3 (5.7)		
Previous emotional problems n (%)			
Yes	2 (4)	2 (3.8)	.33
No	48 (96)	50 (96.2)	

**Table 2:** Comparison of mean score of anxiety domains by Yale Preoperative Anxiety Scale in control group.

Anxiety domains	SD ± X			*P*
	Pre	Post	*t*-test	
Activity	0.34 ± 0.48	0.34 ± 0.48	0.234	1.000
Vocalization	0.62 ± 0.49	0.64 ± 0.48	0.444	.659
Emotional expressivity	0.50 ± 0.51	0.50 ± 0.51	0,002	1.000
Arousal	0.73 ± 0.45	0.42 ± 0.78	1.429	.159
Use of parents	0.87 ± 0.34	0.83 ± 0.38	0.43	.160
Mean total anxiety score	57.45 ± 17.94	56.50 ± 15.63	0.59	.558

**Table 3:** Comparison of mean score of anxiety domains by Yale Preoperative Anxiety Scale in painting group.

Anxiety domains	SD ± X		*t*-test	*P*
	Pre	Post		
Activity	0.29 ± 0.46	0.17 ± 0.38	1.948	.057
Vocalization	0.48 ± 0.50	0.38 ± 0.49	1218	.229
Emotional expressivity	0.44 ± 0.50	0.31 ± 0.47	1.730	.095
Arousal	0.65 ± 0.48	0.62 ± 0.49	0.531 (51)	.598
Use of parents	0.77 ± 0.42	0.73 ± 0.45	0.629 (51)	.532
Mean total anxiety score	50.77 ± 19.20	42.21 ± 15.86	3.698 (51)	.001

**Table 4:** Comparison of mean score of anxiety domains by Yale Preoperative Anxiety Scale in Interactive games group.

Anxiety domains	SD ± X Pre	Post	*t*-test (*d*)	*P*
Activity	0.32 ± 0.47	0.19 ± 0.39	2.2	.031
Vocalization	0.73 ± 0.49	0.78 ± 0.42	−1.000	.321
Emotional expressivity	0.57 ± 0.50	0.38 ± 0.49	2.82	.007
Arousal	0.81 ± 0.40	0.77 ± 0.42	0.704	.484
Use of parents	0.84 ± 0.36	0.81 ± 0.40	7.04	.484
Mean total anxiety score	57.73 ± 17.60	49.91 ± 13.21	4.8198	.001

**Table 5:** Logistic regression analysis examining role of drawing characteristics in predicting anxiety.

Included painting characteristics	*b*	*S*_*b*_	Wald χ^2^	*P*	OR	95% OR	
						Lower	Upper
A1	−2.641	0.740	12.732	<.001	0.071	0.017	0.304
A3	−1.046	0.634	2.719	.099	0.351	0.101	1.218
A4	1.607	0.656	5.995	.014	4.987	1.378	18.048
A6	1.619	0.684	5.599	.018	5.050	1.321	19.315
A9	−2327	0.697	11.159	.001	0.098	0.025	0.382
A13	3.050	0.991	9.482	.002	21.120	3.030	147.185
A17	0.901	0.604	2.226	.136	2.463	0.754	8.049
A19	2.295	0.742	9.566	.002	9.922	2.318	42.478
A25	−3227	0.909	12.599	<.001	0.040	0.007	0.236
Constant	1.202	0.821	2.143	.143	3.326		

OR = odds ratio.

### 3.2. Comparison of anxiety scores at different time intervals between the 2 groups

Patients in the mapping group had significantly lower anxiety scores at treatment room preparation than controls (Ts) [51.9 (23.6) vs 67.2 (22.0)]; mean difference 15.3; 95% confidence interval = 6.4–24.1; *P* = .001] and at post-hospitalization treatment (T1) [27.8 (7.6) vs 33.2 (13.6)]; mean difference 5.3; 95% confidence interval  = 0.93–9.8; *P* = .018). The differences in anxiety levels between the 2 groups at the other time points of observation, such as before the treatment room clinic (T1), waiting area (T2), separation from parents to the treatment room (T3), and entry into the treatment room (T4), were not statistically significant (*P*-values of .584, .335, .228, and .137, respectively). In addition, we analyzed the changes in observed anxiety levels in each group along the 6 time points. Overall, the mYPAS-SF scores of the illustrated group decreased from point 0 at point T, whereas they increased in the control group. Then, from T to T, the anxiety levels of both groups tended to increase. In particular, mYPAS-SF scores increased slightly at T4 compared to T3 and sharply at T5 compared to T4.

### 3.3. Comparison of treatment adherence and quality of life between the 2 groups

We compared the 2 groups in terms of treatment adherence and quality of life scores, which were significantly higher in the painting group compared to the control group (*P* < .05). In particular, the painting group had the highest quality of life scores 3 months after the initial treatment.

### 3.4. Relationship between patients’ drawing characteristics and anxiety states

A logistic regression model (α_in_ = 0.10, α_out_ = 0.15) was developed using the SAS results as the dependent variable, with the drawing group set at 1 and the control group at 0, and the 12 drawing characteristics (two categorical variables) selected by univariate analyses as the independent variables. A total of 9 drawing characteristics were included in the equation in order to examine the relationship between patients’ drawing therapy characteristics and anxiety. The results of the sum test of the model coefficients showed χ^2^ = 56.982, *P* ≤ .001, indicating that the model of the established logistic regression equation was statistically significant. Based on the regression coefficients shown in Table 5, a regression equation was established for the painting therapy on the anxiety characteristics of the patients. Subsequently, the established regression equation was tested by Nagelkerke *R*^2^ coefficient test of the established regression equation. The *R*^2^ values of S-HTP drawing therapy, mandala drawing therapy, and group drawing art therapy were determined to be 0.492, 0.316, and 0.458, respectively, which is a good fit, indicating that the equation has a good level of explanation and the drawing characteristics are suitable for assessing the anxiety level of ASD patients.

### 3.5. The effect of drawing therapy on patients’ anxiety and validity test

The *R*^2^ values of S-HTP drawing therapy, mandala drawing therapy, and group drawing art therapy were determined to be 0.492, 0.316, and 0.458, respectively, which is a good fit, suggesting that the equation has a good level of explanation and the drawing characteristics are suitable for assessing the anxiety level of ASD patients. In order to verify the validity of the drawing treatment on the anxiety level of the patients, the correlation analysis between the drawing efficacy results and the results of the SAS scale was conducted, and the correlation coefficients of the 3 treatments to the 2 tests were rows *R* = 0.55, *R* = 0.44, and *R* = 0.52 with *R* > 0.40, indicating that the consistency of the drawing efficacy results to the results of the SAS was good. Further consistency test, χ^2^ = 46.25, χ^2^ = 6.12, and χ^2^ = 10.25. χ^2^ > 6.63, *P* < .01. It was found that the S-HTP drawing efficacy and group drawing art therapy results of anxiety in patients with ASD were positively correlated with the results of the SAS scale with good consistency.

## 4. Discussion

The findings of this study demonstrate that structured drawing therapy is an effective non-pharmacological intervention for alleviating preoperative anxiety in children with ASD. Our results not only corroborate the established efficacy of distraction techniques, such as interactive play,^[8,18,24–28]^ but also provide a more nuanced understanding of how different artistic modalities function within this population.

The primary mechanism through which drawing likely exerts its effect is by serving as a dual-purpose tool: it acts as a potent distractor from the threatening medical environment while simultaneously providing a non-verbal channel for emotional expression. This aligns with the theoretical framework that art facilitates healing by enabling expression where words may fail.^[29,30]^ For children with ASD, who often experience communication challenges, this non-verbal outlet may be particularly critical. Through drawing, they can project internal anxieties and fears onto a manageable external object, a process akin to the catharsis described in trauma-focused art and play therapy.^[31]^ Therefore, our findings suggest that the benefit of drawing extends beyond mere distraction, tapping into deeper therapeutic processes of emotional regulation, as evidenced by its positive effects on maladaptive behaviors and anxiety in various clinical studies.^[32–35]^

A pivotal finding of our study is the differential effectiveness of various drawing therapies. The significant association between S-HTP drawing and reduced anxiety levels suggests its utility as both a therapeutic and a screening tool. Its unstructured nature may allow for a more direct projection of a child’s inner state, facilitating the expression and catharsis of emotions.^[36–39]^ In contrast, the Mandala technique, which is more structured and centering, did not show a significant correlation with anxiety reduction in our cohort. This could imply that the imposed structure of a Mandala might be less effective for, or even restrictive to, children with ASD who require a more open-ended medium for expression. The effectiveness of group drawing therapy increasing with participant number is another critical insight. It underscores the potential social dimension of therapy for ASD, where a larger group might provide a sense of collective engagement and reduced social pressure,^[40]^ creating a supportive atmosphere that mitigates the anxiety prevalent in more intimate, performance-focused settings.^[41]^

When comparing our drawing intervention to the interactive play control group, the superior reduction in anxiety at key stressful moments (preparation and pre-anesthetic visits) warrants consideration. While play therapy is undoubtedly effective and of high therapeutic value for hospitalized children,^[27,28,42]^ drawing may offer a unique advantage for children with ASD. It is a less socially demanding activity that minimizes the potential for overstimulation – a common challenge in this population. The solitary yet creative act of drawing may provide a “safe harbor” for self-regulation, potentially explaining its enhanced efficacy in the high-stress preoperative context. This is supported by the concept that for children, activities like drawing are not merely pleasurable but a fundamental way of expressing anxiety and fear,^[39]^ and when used as a preparatory educational tool, such as through picture books, it can foster a sense of control and improve cooperation.^[43,44]^

This study has several limitations. First, although multivariate logistic regression was performed to adjust for certain influencing factors, potential confounding variables such as children’s intelligence levels, the severity of ASD symptoms, family educational background, and socioeconomic status were not fully controlled. These factors may have influenced the observed anxiety levels and treatment outcomes. Future studies should include more comprehensive baseline assessments and stratified analyses to better account for these variables. Additionally, the study sample was limited to one region, and the sample size, although adequately powered, may restrict the generalizability of the findings.

## 5. Conclusions

In conclusion, this study demonstrates that structured drawing therapy, particularly the S-HTP technique, is an effective intervention for reducing preoperative anxiety in children with ASD. The differential effectiveness of various drawing modalities suggests that the choice of art-based intervention should be tailored to the individual’s needs, with S-HTP serving as a potent tool for both anxiety screening and alleviation. This study has several limitations. The real-world clinical setting introduced variability in treatments and nursing care, which were not controlled. Furthermore, we did not assess how individual characteristics of the children, such as ASD symptom severity or verbal ability, might influence their response to different drawing therapies. Future research should focus on identifying patient-specific factors that predict treatment success and investigate the long-term benefits of integrating these art-based interventions into standard clinical pathways for children with ASD.

## Author contributions

**Conceptualization:** Jinghong Yao, Qiushi Wang, Yang Qiao, Heyong Shen.

**Data curation:** Jinghong Yao, Qiushi Wang, Yang Qiao, Heyong Shen.

**Formal analysis:** Jinghong Yao, Qiushi Wang, Yang Qiao, Ying Cai, Heyong Shen.

**Funding acquisition:** Qiushi Wang, Heyong Shen.

**Investigation:** Ying Cai, Heyong Shen.

**Writing – original draft:** Jinghong Yao, Ying Cai, Heyong Shen.

**Writing – review & editing:** Jinghong Yao, Yang Qiao, Ying Cai, Heyong Shen.

## References

[1] Reyhani T Dehghan Z Shojaeian R Asgharinekah SM BehnamVashani HR. The influence of the puppet Red Haton preoperative anxiety among hospitalized children with appendicitis in Dr. Shaikh Hospital of Mashhad. Evid Base Care. 2014;4:77–86.

[2] Fortier MA Del Rosario AM Martin SR Kain ZN. Preoperative anxiety in children. Paediatr Anaesth. 2010;20:18–22. 10.1111/j.1460-9592.2010.03263.x 20199609

[3] Kain ZN Mayes LC Caldwell-Andrews AA Karas DE McClain BC. Preoperative anxiety, postoperative pain, and behavioral recovery in young children undergoing surgery. Pediatrics. 2006;118:651–8. 16882820 10.1542/peds.2005-2920

[4] Uddin I Kurkuman AR Jamil T Iftikhar R. Pre-operative anxiety in patients admitted for elective surgery in King Saud Hospital, Unaizah, Al-Qassim, Kingdom of Saudi Arabia. Pak J Med Sci. 2002;18:306–10.

[5] Aytekin A Doru O Kucukoglu S. The effects of distraction on preoperative anxiety level in children. J PeriAnesthesia Nurs. 2016;31:56–62. 10.1016/j.jopan.2014.11.016 26847781

[6] de Moura LA Dias IMG Pereira LV. Prevalence and factors associated with preoperative anxiety in children aged 5–12 years. Rev Latino-Am Enferm. 2016;24:1–7, e2708. 10.1590/1518-8345.0723.2708 PMC4916978 27305179

[7] Lilik Lestari MP Wanda D Hayati H. The effectiveness of distraction (cartoon-patterned clothes and bubble-blowing) on pain and anxiety in preschool children during venipuncture in the emergency department. Compr Child Adolesc. Nurs. 2017;40:22–8. 29166202 10.1080/24694193.2017.1386967

[8] Weber FS. The influence of playful activities on children’s anxiety during the preoperative period at the outpatient surgical center. Jornal de pediatria. 2010;86:209–14. 20419272 10.2223/JPED.2000

[9] Hosseinpour M Memarzadeh M. Use of a preoperative playroom to prepare children for surgery. Eur J Pediatr Surg. 2010;20:408–11. 21058244 10.1055/s-0030-1265172

[10] Anes L Obi M. Hospital clowning as play stimulus in healthcare. Children (Basel). 2014;1:374–89. 27417485 10.3390/children1030374 PMC4928740

[11] Zarezadeh Kheibari S Hokm Abadi ME Mohebi Anabat A Shakiba SH Hosseini Largany SF. The effectiveness of expressive group art therapy on decreasing anxiety of orphaned children. J Pract Clin Psychol (J.P.C.P). 2014;2:134–42.

[12] Gholamzade Khadar M Babapour J Sabouri Moghaddam H. The effect of art therapy based on painting therapy in reducing symptoms of the oppositional defiant disorder (ODD) in elementary school boys. Proc Soc Behav Sci. 2013;84:1872–8.

[13] Kain ZN Mayes LC O’Connor TZ Cicchetti DV. Preoperative anxiety in children. Predictors and outcomes. Arch Pediatr Adolesc Med. 1996;150:1238–45. 8953995 10.1001/archpedi.1996.02170370016002

[14] Hashemian P Jarahi L. Effect of painting therapy on aggression in educable intellectually disabled students. Psych. 2014;05:2058–63.

[15] Safaria T Yunita A. The efficacy of art therapy to reduce anxiety among bullying victims. Int J Res Stud Psych. 2014;3:77–88.

[16] Zarei B Valiee S Khosravi F Fathi M. The effect of the multimedia-based nursing visit on preoperative anxiety and vital signs in patients undergoing lumbar disc herniation surgery: a randomized clinical trial. J Perioperat Pract. 2018;28(:7–15. 10.1177/1750458917742045 29376786

[17] Kain ZN Caldwell-Andrews AA Krivutza DM. Interactive music therapy as a treatment for preoperative anxiety in children: a randomized controlled trial. Anesth Analg. 2004;98:1260–6, table of contents. 15105197 10.1213/01.ane.0000111205.82346.c1

[18] Gao XL Liu Y Tian S Zhang DQ Wu QP. Effect of interesting games on relief of preoperative anxiety in preschool children. Int J Nurs Sci. 2014;1:89–92.

[19] Aroonpruksakul N Nithiuthai J Jenjarat S Choorerk D. Validation and reliability of full and short Thai-version modified Yale preoperative anxiety scale (m-YPAS) in young children. J Med Assoc Thai. 2017;100:53–8.

[20] Vaezzadeh N Douki ZE Hadipour A Osia S Shahmohammadi S Sadeghi R. The effect of performing preoperative preparation programs on school-age children’s anxiety. Iran J Pediatr. 2011;21:461–6. 23056832 PMC3446145

[21] Ghabeli F Moheb N Nasab SDH. Effect of toys and preoperative visit on reducing children’s anxiety and their parents before surgery and satisfaction with the treatment process. J Caring Sci. 2014;3:21–8. 25276745 10.5681/jcs.2014.003 PMC4134164

[22] Al-Yateem N Brenner M Shorrab AA Docherty C. Play distraction versus pharmacological treatment to reduce anxiety levels in children undergoing day surgery: a randomized controlled non-inferiority trial. Child Care Health Dev. 2016;42:572–81. 27080806 10.1111/cch.12343

[23] He HG Zhu L Chan SWC. Therapeutic play intervention on children’s preoperative anxiety, negative emotional manifestation and postoperative pain: a randomized controlled trial. J Adv Nurs. 2015;71:1032–43. 25561079 10.1111/jan.12608

[24] El-Moazen AE Mohamed SA Kareem MA. Effect of selected play activities on preoperative anxiety level and fear among children undergoing abdominal surgeries. Egypt Nurs J. 2018;15:205–16.

[25] Silva RD Austregesilo SC Ithamar L Lima LS. Therapeutic play to prepare children for invasive procedures: a systematic review. Jornal de pediatria. 2017;93:6–16. 27485756 10.1016/j.jped.2016.06.005

[26] Hockenberry M.J. Wilson D. Wong’s Essentials of Pediatric Nursing, eighth ed. Elsevier Mosby, St. Louis. 2009.

[27] Ramdaniati S Hermaningsih S Muryati N. Comparison study of art therapy and play therapy in reducing anxiety on pre-school children who experience hospitalization. Open J Nurs. 2016;6:46–52.

[28] Handajani D Yunita N. Apakah ada Pengaruh Terapi Bermain puzzle terhadap tingkat Kecemasan Anak Usia Prasekolah Yang Mengalami Hospitalisasi Di Rs Bhakti Rahayu Surabaya. J Manaj Kesehat Indones. 2019;7:198–204.

[29] Regev D Cohen-Yatziv L. Effectiveness of art therapy with adult clients in 2018 – what progress has been made. Front Psychol. 2018;9:1531. 30210388 10.3389/fpsyg.2018.01531 PMC6124538

[30] Abbing A Ponstein A vanHooren S deSonneville L Swaab H Baars E. The effectiveness of art therapy for anxiety in adults: a systematic review of randomized and non-randomized controlled trials. PLoS One. 2018;13:e0208716. 30557381 10.1371/journal.pone.0208716 PMC6296656

[31] Bazargan Y Pakdaman S. The effectiveness of art therapy on reducing internalizing and externalizing problems of female adolescents. Arch Iran Med. 2016;19:51–6. 26702749

[32] Yan H Chen J Huang J. School bullying among left-behind children: the efficacy of art therapy on reducing bullying victimization. Front Psychiatr. 2019;10:40. 10.3389/fpsyt.2019.00040 PMC6374290 30792670

[33] Sadeghi N Raeisi L. Effect of drawing distraction on children’s preoperative anxiety: a clinical trial. J Mazandaran Univ Med Sci. 2019;29:86–97.

[34] Wowiling FE Ismanto AY Babakal A. Pengaruh Mewarnai Gambar Terhadap tingkat Kecemasan Pada Anak Usia Pra Sekolah Yang Mengalami Hospitalisasi Di Ruang Irina E. RSUP. BLU. Prof. Dr. R.D. Kandao, Menado. J Keperawatan. 2014;2:1–8.

[35] Pravitasari A Warsito BE. Perbedaan Tingkat Kecemasan Pasien Anak Usia Pra Sekolah Sebelum dan sesudah Proses Mewarnai. J Keperawatan Diponegoro. 2012;1:16–21.

[36] Kapti RE Ahsan A Istiqomah A. Pengaruh Bermain Dengan Mewarnai Terhadap Penurunan Skor Perilaku mal Adaptif Anak Usia Pra Sekolah (3-5 tahun) Yang Mengalami Hospitalisasi Di RS. Kabupaten. Kediri. J Ilmu Keperawatan. 2013;1:169–75.

[37] Stuckey HL Nobel J. The connection between art, healing, and public health: a review of current literature. Am J Public Health. 2010;100:254–63. 20019311 10.2105/AJPH.2008.156497 PMC2804629

[38] Lantz J Raiz L. Play and art in existential trauma therapy with children and their parents. Contemp Family Ther. 2003;25:165–77.

[39] Armstrong TS Aitken HL. The developing role of play preparation in pediatric anesthesia. Paediatr Anaesth. 2000;10:1–4. 10632902 10.1046/j.1460-9592.2000.00406.x

[40] Ghadampour E Amirian L Radpour F. The effect of group painting therapy on loneliness, control of anger, and social adjustment of primary school students. J Child Ment Health. 2019;6:119–31.

[41] West N Christopher N Stratton K Gorges M Brown Z. Reducing preoperative anxiety with child lifepreparation prior to intravenous induction of anesthesia: a randomized controlled trial. Paediatr Anaesth. 2020;30:168–80. 31869478 10.1111/pan.13802

[42] Ryu JH Park SJ Park JW. Randomized clinical trial of immersive virtual reality tour of the operating theatre in children before anaesthesia. Br J Surg. 2017;104:1628–33. 28975600 10.1002/bjs.10684

[43] Bijttebier P Vertommen H. The impact of previous experience on children’s reactions to venepunctures. J Health Psychol. 1998;3:39–46. 22021341 10.1177/135910539800300103

[44] Hui WJ Pikkarainen M Nah SA. parental experiences whilewaiting for children undergoing surgery in Singapore. J Pediatr Nurs. 2020;52:e42–50. 31983480 10.1016/j.pedn.2020.01.004

